# Prevalence of Maxillary and Mandibular Frenal Attachment and Its Association with Age, Gender, and Oral Hygiene Status in Nepalese Population Seeking Dental Treatment

**DOI:** 10.1155/2024/4870253

**Published:** 2024-01-11

**Authors:** Arjun Hari Rijal, Manoj Humagain, Simant Lamichhane, Pratistha Ghimire, Sachita Thapa

**Affiliations:** ^1^Department of Periodontology and Oral Implantology, Kathmandu University School of Medical Sciences, Dhulikhel, Kavrepalanchok, Nepal; ^2^Dental Department, Methinkot Hospital, Bhakundebesi, Kavrepalanchok, Nepal

## Abstract

**Objective:**

Frenum attachments are folds of mucous membrane that connect the lips to the alveolar mucosa and underlying periosteum. Aberrant positioning of the maxillary and mandibular labial frenum can lead to various clinical issues, including mucogingival problems and midline diastema. The objective of this study was to assess the prevalence of maxillary and mandibular frenal attachments and their association with age, gender, and oral hygiene status in the Nepalese population seeking dental treatment.

**Materials and Methods:**

This descriptive cross-sectional study was conducted over a period of 6 months, from February 2023 to August 2023, among patients visiting the Department of Periodontology and Oral Implantology, aged 6 years and above, after obtaining official permission from the Institutional Review Committee (IRC-KUSMS Approval No. 8/23). The study collected data on morphological variations of frenal attachment and various periodontal parameters such as Oral Hygiene Index-Simplified (OHI-S), pocket depth, recession, and midline diastema in both arches. Descriptive statistics, including frequency and percentage, were used to analyze the data. The *χ*^2^-test was employed to determine the correlation between gender and types of frenulum attachment, while analysis of variance was used to assess the association of frenal attachments with oral hygiene status.

**Results:**

Among 380 patients, the prevalence of frenal attachment was as follows: gingival 248 (65.30%), mucosal 71 (18.70%), papillary 42 (11.10%), and papillary penetrating 19 (5.00%) in the maxilla, and mucosal 225 (59.20%) and gingival 155 (40.78%) in mandible. Among the different morphological variations of frenal attachments, normal frenum was the most common, accounting for 231 cases (60.80%), followed by frenum with a nodule, with 101 cases (26.60%).

**Conclusions:**

The study found significant associations between frenal attachment and gender, as well as oral hygiene status. The prevalence of frenal attachments in this study was comparable to findings from previous research.

## 1. Introduction

The frenum is an anatomical structure found in the oral cavity that exhibits significant variability. It can be described as a “fibrous band of tissue attached to the mandible and maxillae bones, situated superficially to muscle attachments” [1]. The prominent frena in the normal oral cavity are the maxillary/mandibular labial frena and the lingual frenum [2]. Among the various frena, the maxillary frena undergo dynamic changes throughout different stages of human growth and development [3].

Depending upon the extension of attachment of fibers, frena have been classified as [4] mucosal—when the frenal fibers are attached up to mucogingival junction, gingival—when fibers are inserted within attached gingiva, papillary—when fibers are extending into interdental papilla, and papilla penetrating—when the frenal fibers cross the alveolar process and extend up to palatine papilla.

Sewerin [5] has also classified the variations of frenum as: normal frenum, normal frenum with a nodule, normal frenum with appendix, normal frenum with nichum, bifid labial frenum, persistent tectolabial frenum, double frenum, and wider frenum.

Frenal attachments that encroach on the gingiva cause distension, plaque buildup, accelerated periodontal recession, and treatment recurrence [6]. Therefore, early identification prevents complications.

However, relatively few studies have been documented pertaining to the prevalence and types of the labial frenum in both maxillary and mandibular arch. Thus, the aim of this study was to evaluate the prevalence of frenal variations and its association with age, gender, and oral hygiene status in a diverse ethnic population.

## 2. Materials and Methods

A descriptive cross-sectional study was carried out from February 2023 to August 2023, following the acquisition of ethical clearance from the Institutional Review Committee (IRC) of Kathmandu University School of Medical Sciences (IRC KUSMS, IRC number: 8/23). All patients visiting the Department of Periodontology and Oral Implantology, KUSMS, were included in the study after obtaining written informed consent. For adult participants, written informed consent was obtained directly, while in the case of minors, the examination was conducted in the presence of their parents, and written informed consent was obtained from them.

Convenience sampling was used to collect the study samples, and sample size was calculated using data from the study by Chaulagain et al. [6]:

(1) $$\begin{array}{c} \begin{array}{c} n=z^{2}p\left(1-p\right)/d^{2}\\ n=1.96\times 1.96\times 45.1\times \left(100-45.1\right)/5\times 5=380=380, \end{array} \end{array}$$

where *z* = 1.96 at 95% confidence interval; *p* is the prevalence; *d* is the absolute margin of error to be tolerated; and *p* is the maximum prevalence of mucosal frenal attachment taken from a study by Chaulagain et al. [6] = 45.1%.

All patients aged 6 years and above, visiting the Department of Periodontology and Oral Implantology, were included in the study. The exclusion criteria consisted of patients who had previously undergone surgery on the upper and lower labial frenum, a history of trauma or injuries in the maxillary and mandibular anterior region, any congenital or developmental abnormality in the upper and lower frenum, a past history of orthodontic treatment or undergoing current orthodontic treatment, and patients taking medication known to affect the gingiva.

**Figure 1:**
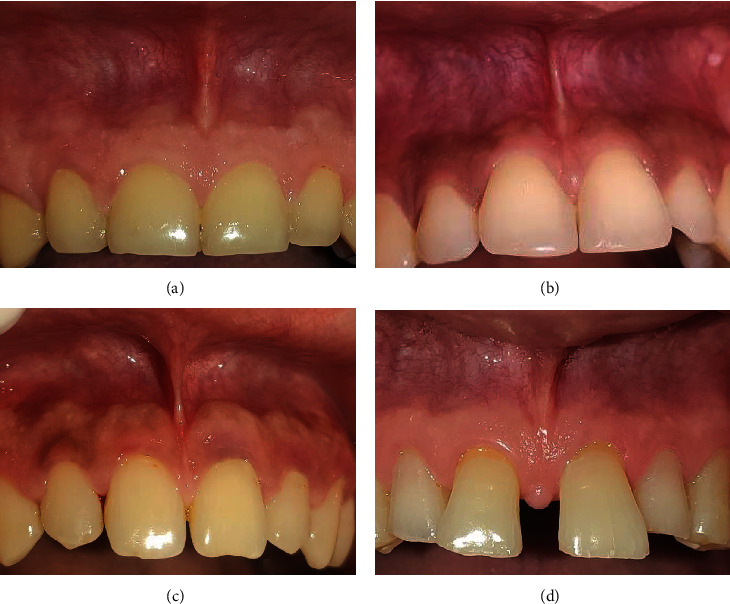
Placek Mirko's classification of frenal attachment (a) mucosal, (b) gingival, (c) papillary, and (d) papillary penetrating.

**Figure 2:**
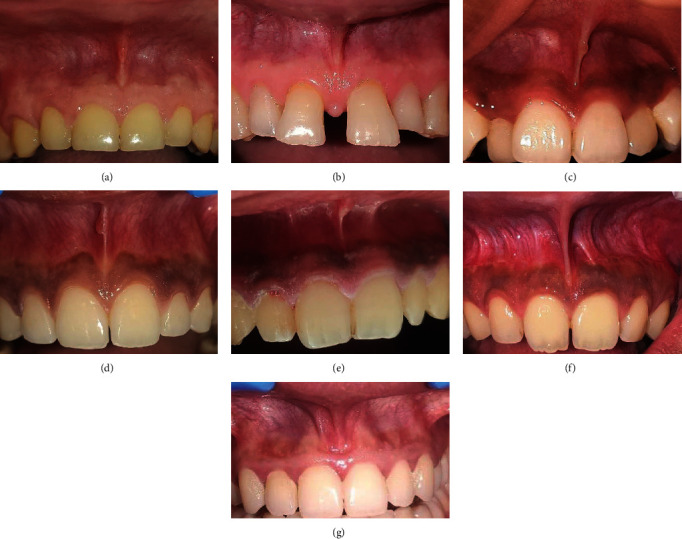
Morphological variations of frenal attachment according to Sewerin's [5] study: (a) normal frenum, (b) persistent tectolabial frenum, (c) frenum with appendix, (d) frenum with nodule, (e) duplication of frenum, (f) recess of frenum, and (g) bifid frenum.

Interested participants who provided informed consent underwent examination by a single examiner. The examiner raised the lip in upward/downward and outward directions to visualize the frenum and took photographs for classification according to Mirko's (Figure 1) and Sewerin's (Figure 2) classifications. Examination was done for both maxillary and mandibular labial frenal attachment. Additionally, the Oral Hygiene Index-Simplified (OHI-S) was recorded for all indexed teeth using an explorer. Periodontal examinations, including measurements of gingival recession, periodontal pockets, midline diastema, and the width and thickness of attached gingiva, were also performed.

Periodontal pocket = measured from gingival margin to base of the pocket.

Gingival recession = from cementoenamel junction to gingival margin.

Width of attached gingiva = examined as adequate and inadequate by using tension test (by stretching lips in upward/downward and outward direction).

Thickness of attached gingiva = by probe transparency methods (probe visible = thin biotype, probe not visible = thick biotype).

The data were entered and analyzed statistically using IBM Corp.'s Statistical Package for the Social Sciences Statistics for Windows, version 21.0. A confidence level of 95% (*p*-value < 0.05) was employed to assess significance. Descriptive statistics, such as frequency and percentage, were utilized to examine the data. The *χ*^2^-test was employed to assess the correlation between gender groups and frenulum attachment types, while analysis of variance (ANOVA) was utilized to investigate the association between age groups and various frenulum attachments.

## 3. Results

In the current study, a total of 380 patients were enrolled. Among them, 179 (47.10%) were male, and the remaining 201 (52.90%) were female. The mean age of the participants was 33.58 ± 14.28 years. All the demographic data and clinical parameters are summarized in Table 1. Notably, more than 95% of the participants exhibited the absence of gingival recession, midline diastema, and periodontal pocket, indicating favorable oral health conditions among the majority of the subjects.

Regarding the thickness of gingiva, it was found that thick gingiva was more prevalent in the maxilla, with 253 (66.60%) participants exhibiting this characteristic. On the other hand, thin gingiva was more commonly observed in the mandible, with 200 (52.60%) participants showing this trait. Moreover, the width of attached gingiva was adequate in both jaws for most of the cases, indicating overall positive gingival health.

The study also assessed the prevalence of frenal attachment according to the classification by Placek Mirko, both in the maxilla and mandible. In the maxilla, the most prevalent type of frenal attachment was gingival, present in 248 (65.30%) participants. This was followed by mucosal attachment in 71 (18.70%), papillary attachment in 42 (11.10%), and papillary penetrating attachment in 19 (5.00%) participants. In contrast, in the mandible, the most common frenal attachment type was mucosal, observed in 225 (59.20%) participants, followed by gingival attachment in 155 (40.78%) participants. Notably, papillary and papillary penetrating frenal attachments were absent in the mandible for the subjects in the current study, as shown in Table 2.

In this study, we examined the variation in frenal attachment using Sewerin's classification in both the maxilla and mandible. In the maxilla, the most frequent type of frenal attachment was the normal frenum, observed in 231 (60.80%) cases, followed by the frenum with a nodule in 101 (26.60%) cases. The duplication of frenum was the least prevalent, found in only one case (0.30%). In contrast, the mandible showed fewer morphological variations, with the normal frenum being the most common type, present in 358 (94.20%) cases. The duplication of frenum was seen in 20 (5.30%), and the bifid frenum was observed in two (0.50%) cases (Table 2).

Additionally, we utilized the Placek Mirko system to investigate the prevalence of gingival and mucosal frenal attachment in both genders (male and female) within the maxilla and mandible. In the maxilla, gingival frenal attachment was more prevalent in both males, with 106 (27.89%) cases, and females, with 142 (37.13%) cases. Conversely, mucosal frenal attachment was most common in the mandible, with 124 (32.63%) cases in males and 101 (26.57%) cases in females. We employed the *χ*^2^-test to assess the association between gender and frenal attachment types, and the results showed statistical significance (maxilla *p*-value = 0.033, mandible *p*-value ≤ 0.001) (Table 3).

The distribution of frenal attachment according to both systems in the maxilla and mandible based on age was provided in Table 4. To assess the relationship between frenal attachment (Placek Mirko) and oral hygiene status, we employed ANOVA (Table 5). In the mandible, the results showed a statistically significant association (*p*-value ≤ 0.001). Further, a post hoc test was conducted to explore any additional associations, and it revealed statistically significant results (detailed data are not shown in the table).

## 4. Discussion

The frenum is a component of the mucous membrane, taking the form of a fibrous collagenous band, that serves to connect the lips to the alveolar mucosa, gingiva, and the underlying periosteum both in the labial and lingual regions [7, 8]. Typically found in the maxillary labial/buccal, mandibular labial, and mandibular lingual areas, the frenum's attachment level and morphological shape can vary [9]. One positive function of the frenum is its role in stabilizing the upper and lower lips as well as the tongue in the floor of the mouth [9]. It is regarded as a residual structure that remains after a certain process, representing the connection between the upper lip's tubercle and the palatine papilla [8]. Initially, the frenal attachment is higher in the middle of the attached gingiva, but as development progresses, it shifts more apically below the mucogingival junction [10]. Histologically, the frenum is comprised of parts of the epithelium, connective tissues, and various fibers of skeletal muscles [11, 12].

The location of frenal attachment within the oral cavity can vary. A widely accepted classification, proposed by Mirko et al. [4], categorizes frenal attachment into four types: mucosal, gingival, papillary, and papillary penetrating. Mucosal and gingival attachments are considered normal, while papillary and papillary penetrating attachments are regarded as aberrant forms of the frenum [13]. In Mirko's et al. [4] classical study, the maxilla predominantly exhibited gingival (46.5%) and mucosal (34.3%) attachments, while the mandible predominantly showed mucosal attachment (92.1%). In our present study, the prevalence of maxillary frenal attachments was as follows: gingival (65.30%), mucosal (18.70%), papillary (11.10%), and papillary penetrating (5.00%). These findings align with previously published literature on the subject [7, 14–17]. However, it is worth noting that some studies in the literature differ from our present study, reporting a higher prevalence of mucosal frenal attachments compared to gingival attachments (Table 6).

The differences in the prevalence of frenal attachments among various studies may be attributed to several factors, such as variations in sample population size, differences in inclusion and exclusion criteria, ethnic backgrounds of the participants, age groups studied, and the proficiency of examiners in their evaluations.

Likewise, in the mandible, mucosal frenal attachment was found to be more prevalent, accounting for 225 (59.20%) cases, followed by gingival attachment with 155 (40.78%) cases. However, papillary and papillary penetrating frenal attachments were not present in the current sample population. These findings closely mirror the results obtained in the classic study conducted by Mirko et al. [4].

In this study, we also examined the morphological variations of frenal attachment in the maxillary anterior labial region using the classification provided by Sewerin [5]. The findings revealed that the most prevalent morphological variations in the maxilla were normal frenum, accounting for 231 cases (60.80%), and frenum with a nodule, accounting for 101 cases (26.60%). These results align with several other studies that reported similar findings [6, 17, 19–21, 23, 25, 26, 32]. Whereas in some studies frenum with appendix, [5, 16, 31] persistent tectolabial frenum, [17] were most prevalent frenal attachment. Moving on to the mandibular anterior region, our investigation revealed only three variations in frenal attachment: normal frenum, comprising 358 (94.20%) cases, duplication of frenum, with 20 (5.30%) cases, and bifid frenum, with two (0.50%) cases. Interestingly, during our literature review, we were unable to find similar studies that utilized the Sewerin classification system for the mandible, making direct comparisons impossible.

The association between frenal attachment and gender showed significance in both the maxilla and mandible when using Mirko et al. [4] classification system, with *p*-values of 0.033 and ≤0.001, respectively. In the case of the Sewerin system, a significant association was observed only in the mandible, with a *p*-value of 0.002. These findings are consistent with some previous studies [4]. In contrast to our current study, no association between gender and frenal attachment was reported in one study [18].

Initially, the frenum is situated at a higher position, attached to the gingiva. As the jaw develops, it tends to shift in the apical direction and typically reaches the alveolar mucosa [8]. However, in our present study, this concept was not clearly evident, which aligns with the findings of another study [18]. It is possible that the results obtained in our study may be attributed to the relatively small sample size, particularly in older age groups.

The position of different types of frenal attachment is closely linked to various periodontal, gingival, and esthetic issues. High frenal attachments, such as papillary and papillary penetrating attachments, are particularly associated with these problems [4]. In the case of papillary frenal attachment, it is usually located in the interdental papilla at the midline. On the other hand, papillary penetrating attachment involves the frenum extending right up to the papilla while inserting into the attached gingiva [4]. These two types of frenal attachments can lead to several challenges, including difficulties with speech, esthetic concerns, mastication problems, and the development of diastema. Moreover, highly placed aberrant frenal attachment can make it challenging for patients to maintain proper oral hygiene practices, resulting in various periodontal issues such as pockets and gingival recessions [18].

In our present study, we aimed to investigate any potential association between frenal attachments and oral hygiene status. The results revealed a strong correlation between frenal attachments and oral hygiene status (*p*-value ≤ 0.001). However, in the case of maxillary arches, the association was not statistically significant (*p*-value = 0.085). Reason behind this may be the prevalence of mucosal and gingival frenal attachment is more as compared to papillary and papillary penetrating. Also, in maxilla compromised oral hygiene status is usually associated with lack of adequate width of attached gingiva.

Furthermore, we observed that gingival recession, thin gingival biotype, and inadequate width of attachment were more pronounced in the mandible [33]. This could explain the robust link between frenal attachment and oral hygiene status in the mandible. A similar finding was reported in another study [34]. These findings underscore the crucial importance of maintaining proper oral hygiene practices [35, 36] in patients with aberrant frenal attachment. Neglecting oral hygiene in such cases may lead to a heightened risk of progressing periodontal diseases.

Similarly, specific types of frenal attachments or the complete absence of frenal attachments have been associated with various syndromes. Some of these syndromes include Ehlers–Danlos syndrome, infantile hypertrophic pyloric stenosis, holoprosencephaly, Ellis-van Creveld syndrome, and orofacial-digital syndrome [8, 9]. Therefore, the presence or absence of certain frenal attachments in conjunction with syndromic manifestations can serve as diagnostic indicators in many cases.

To address the issues associated with aberrant frenal attachments, frenotomy or frenectomy can be performed using different surgical techniques. Frenectomy can be carried out using scalpel techniques, electrosurgery, or lasers [18, 37, 38]. Electrosurgery and laser techniques offer several advantages over conventional scalpel methods, including reduced bleeding during and after the surgery, minimal patient discomfort, and smooth healing with less scar formation [39].

## 5. Conclusion

The morphology and attachment of the labial frenum in the maxilla and mandible exhibit considerable variation. Within the scope of this study, it was observed that in the maxilla, gingival followed by mucosal attachment was more prevalent, whereas in the mandible, mucosal followed by gingival attachment was more commonly found. Moreover, normal frenum and frenum with a nodule were the most frequently encountered types of frenal attachment in the present study.

Furthermore, a significant association was found between frenal attachment and gender, while no such association was observed between age and frenal attachment. Additionally, an association was identified between oral hygiene status and frenal attachment in the mandible. These findings underscore the importance of maintaining good oral hygiene practices, particularly in cases with high (aberrant) frenal attachment, especially in papillary and papillary penetrating situations.

Given these results, it is imperative to conduct comprehensive clinical examinations and carefully evaluate the position of the frenum during routine clinical practice. By doing so, we can potentially reduce the occurrence of periodontal problems associated with high frenal attachment.

## Figures and Tables

**Table 1:** Distribution of various parameters.

Parameters	Distribution, *n* (%)	
Gender		
Male	179 (47.10)	
Female	201 (52.90)	
Age		
<20 years	33 (8.68)	
21−40 years	235 (61.84)	
41−60 years	94 (24.73)	
>60 years	15 (3.95)	
Gingival recession	Maxilla	Mandible
Present	18 (4.70)	61 (16.10)
Absent	362 (95.30)	319 (83.90)
Midline diastema	Maxilla	Mandible
Present	39 (10.30)	5 (1.30)
Absent	341 (89.70)	375 (98.70)
Periodontal pocket	Maxilla	Mandible
Present	3 (0.80)	3 (0.80)
Absent	377 (99.20)	377 (99.20)
Width of attached gingiva	Maxilla	Mandible
Adequate	380	369 (97.10)
Inadequate	—	11 (2.90)
Thickness of gingiva	Maxilla	Mandible
Thin	127 (33.40)	200 (52.60)
Thick	253 (66.60)	180 (47.4)

**Table 2:** Distribution of frenal attachment in maxilla and mandible.

Prevalence of frenal attachment according to Mirko's classification		
Types of frenum	Maxillary frenal attachment, *n* (%)	Mandibular frenal attachment
Mucosal	71 (18.70)	225 (59.20)
Gingival	248 (65.30)	155 (40.78)
Papillary	42 (11.10)	0 (0.00)
Papillary penetrating	19 (5.00)	0 (0.00)
Total	380	380

Prevalence of frenal attachment morphology according to Sewerin's classification		

Normal frenum	231 (60.80)	358 (94.20)
Persistent tectolabial frenum	10 (2.60)	0
Frenum with appendix	32 (8.40)	0
Frenum with nodule	101 (26.60)	0
Duplication of frenum	1 (0.30)	20 (5.30)
Recess of frenum	2 (0.50)	0
Bifid frenum	3 (0.80)	2 (0.50)
Total	380	380

**Table 3:** Distribution of frenal attachment in maxilla and mandible based on gender.

Prevalence of frenal attachment according to Mirko's classification						
Types of frenum	Maxillary frenal attachment		*p*-Value	Mandibular frenal attachment		*p*-Value
	Male	Female	0.033	Male	Female	** **≤0.001
Mucosal	35 (19.60)	36 (17.90)		124 (69.30)	101 (50.20)	
Gingival	106 (59.20)	142 (70.60)		55 (30.80)	100 (49.80)	
Papillary	28 (15.60)	15 (7.00)		0 (0.00)	0 (0.00)	
Papillary penetrating	10 (5.60)	9 (4.50)		0 (0.00)	0 (0.00)	
Total				179	201	

Prevalence of frenal attachment morphology according to Sewerin's classification						

Normal frenum	111	120	0.296	161	197	0.002
Persistent tectolabial frenum	5	5		0	0	
Frenum with appendix	18	14		0	0	
Frenum with nodule	44	57		0	0	
Duplication of frenum	1	0		17	3	
Recess of frenum	0	2		0	0	
Bifid frenum	0	3		1	1	
Total						

*χ* ^2^-test, *p*  < 0.05 is significant.

**Table 4:** Distribution of frenal attachment in maxilla and mandible based on age.

Prevalence of frenal attachment according to Mirko's classification										
Types of frenum	Maxillary frenal attachment				Total, *n* (%)	Mandibular frenal attachment				Total, *n* (%)
	<20 years	21−40 years	41−60 years	>60 years		<20 years	21−40 years	41−60 years	>60 years	
Mucosal	8	50	11	2	71 (18.70)	18	127	71	9	225 (59.20)
Gingival	20	152	64	12	248 (65.30)	17	108	23	6	155 (40.80)
Papillary	5	24	13	0	42 (11.10)	0	0	0	0	—
Papillary penetrating	1	10	6	1	19 (5.00)	0	0	0	0	—

Prevalence of frenal attachment morphology according to Sewerin's classification										

Normal frenum	15	159	52	5		34	225	84	215	
Persistent tectolabial frenum	0	15	4	1		0	0	0	0	
Frenum with appendix	4	21	5	2		0	0	0	0	
Frenum with nodule	16	45	33	7		0	0	0	0	
Duplication of frenum	0	1	0	0		1	11	8	0	
Recess of frenum	0	2	0	0		0	0	0	0	
Bifid frenum	0	3	0	0		0	0	2	0	

ANOVA, *χ*^2^-test, *p*  < 0.05 is significant.

**Table 5:** Association of frenal attachment with oral hygiene status (OHI-S).

Prevalence of frenal maxillary attachment according to Mirko's classification				
Frenal attachment	Total number	Oral hygiene status (OHI-S)		Significance
		Mean	Mean standard deviation	0.085
Mucosal	71	2.47	2.47–1.17	
Gingival	248	2.31	2.31–1.00	
Papillary	42	2.07	2.07–1.08	
Papillary penetrating	19	2.70	2.70–0.64	

Prevalence of frenal mandibular attachment according to Mirko's classification				

Mucosal	225	2.50	2.50–1.16	** ≤0.001**
Gingival	155	3.92	3.92–1.98	
Papillary	0	0.00	0.00–0.00	
Papillary penetrating	0	0.00	0.00–0.00	

*χ* ^2^-test, *p*  < 0.05 is significant. Bold means value is statistically significant.

**Table 6:** Prevalence of frenal attachment in various studies which are inconsistent with the present study.

S. no.	Various studies evaluating the prevalence of frenal attachment in maxilla (Placek Mirko's classification)	Population in which study was conducted	Prevalence (%)			
			Mucosal	Gingival	Papillary	Papillary penetrating
1.	Dahal et al. [18]	Nepalese	59.30	32.90	6.40	1.40
2.	Chaulagain et al. [6]	Nepalese	100.00	0.00	0.00	0.00
3.	Pandiyan and Hedge [19]	Malasian	76.00	12.00	7.50	4.50
4.	Rajani et al. [20]	Indian	42.00	34.00	20.00	4.00
5.	Jindal et al. [21]	Indian	66.00	28.40	2.40	3.20
6.	Alwan [22]	Iraqi	37.31	36.32	14.53	2.81
7.	Kotian and Jeevanandan [23]	Indian	67.00	24.50	7.50	1.00
8.	Patel et al. [24]	Indian	56.90	23.70	11.90	7.50
9.	Joshi et al. [25]	Nepalese	60.00	29.70	6.20	4.10
10.	Bowsiya and Arjunkumar [26]	Indian	90.40	8.40	1.20	0.00
11.	Varghese et al. [27]	Indian	42.20	33.33	21.11	3.33
12.	Nandita et al. [28]	Indian	70.00	30.00	0.00	0.00
13.	Rajkarnikar et al. [29]	Nepalese	70.50	28.40	0.80	0.30
14.	Jonathan et al. [30]	Indian	47.50	38.10	0.00	14.20
15.	Sekar et al. [31]	Indian	63.30	26.80	7.20	2.60

## Data Availability

The data used to support the findings of this study are included within the article.

## Additional Points

*Limitations of the Study*. Since this is a cross-sectional study, the level of evidence provided by this study is less as compared to case control and cohort studies. The results of the current study may not be generalized to the entire Nepali population due to ethnic and cultural differences. This sample consisted only of patients visiting the dental college located in Kavrepalanchok, Nepal. Hence, the result cannot be generalized for the entire Nepalese population. Also, only labial frenal attachment was included in the studies. There are various factors besides frenal attachment that could affect the oral hygiene of the patient like position of the tooth in the respective arch, patient's oral hygiene habits, and personal habits, which were not included in the study.
